# Environmental influences on maize-*Aspergillus flavus* interactions and aflatoxin production

**DOI:** 10.3389/fmicb.2014.00040

**Published:** 2014-02-05

**Authors:** Jake C. Fountain, Brian T. Scully, Xinzhi Ni, Robert C. Kemerait, Robert D. Lee, Zhi-Yuan Chen, Baozhu Guo

**Affiliations:** ^1^Department of Plant Pathology, University of GeorgiaTifton, GA, USA; ^2^Crop Protection and Management Research Unit, Agricultural Research Service – United States Department of AgricultureTifton, GA, USA; ^3^Crop Genetics and Breeding Research Unit, Agricultural Research Service – United States Department of AgricultureTifton, GA, USA; ^4^Department of Crop and Soil Sciences, University of GeorgiaTifton, GA, USA; ^5^Department of Plant Pathology and Crop Physiology, Louisiana State UniversityBaton Rouge, LA, USA

**Keywords:** maize, aflatoxin contamination, environment effects, oxidative stress, host resistance

## Abstract

Since the early 1960s, the fungal pathogen *Aspergillus flavus* (Link ex Fr.) has been the focus of intensive research due to the production of carcinogenic and highly toxic secondary metabolites collectively known as aflatoxins following pre-harvest colonization of crops. Given this recurrent problem and the occurrence of a severe aflatoxin outbreak in maize (*Zea mays* L.), particularly in the Southeast U.S. in the 1977 growing season, a significant research effort has been put forth to determine the nature of the interaction occurring between aflatoxin production, *A. flavus, *environment and its various hosts before harvest. Many studies have investigated this interaction at the genetic, transcript, and protein levels, and in terms of fungal biology at either pre- or post-harvest time points. Later experiments have indicated that the interaction and overall resistance phenotype of the host is a quantitative trait with a relatively low heritability. In addition, a high degree of environmental interaction has been noted, particularly with sources of abiotic stress for either the host or the fungus such as drought or heat stresses. Here, we review the history of research into this complex interaction and propose future directions for elucidating the relationship between resistance and susceptibility to *A. flavus* colonization, abiotic stress, and its relationship to oxidative stress in which aflatoxin production may function as a form of antioxidant protection to the producing fungus.

## INTRODUCTION

*Aspergillus flavus* (Link ex Fr.; Teleomorph:* Petromyces flavus*; Horn et al., 2009a,b) is a facultative, plant parasitic pathogen, which has the ability to colonize a number of common crop species including corn, cotton, peanuts, and many other crops (Diener et al., 1987). Economic losses due to the infection of grain crops such as maize (*Zea mays* L.) by *A. flavus* is not primarily due to the expression of symptoms known as *Aspergillus* ear rot but, rather, is due to the subsequent contamination of the grain with the fungal metabolite aflatoxin. Aflatoxins are a group of polyketide-derived furanocoumarin secondary metabolites produced by certain species of fungi, including the genus *Aspergillus* (Bennett and Klich, 2003; Chanda et al., 2009). Aflatoxins are highly carcinogenic and can be acutely toxic or fatal if ingested in sufficient quantities for both livestock and humans (Shephard, 2008).

Although this species was first described by Link in 1809 (Amaike and Keller, 2011), major research on the biology and pathogenicity of *A. flavus* did not commence until the mid 1960s with the incidence of Turkey X disease which killed over 100,000 turkey poults due to aflatoxin contaminated feed associated with *A. flavus* infected peanuts (Wogan, 1966). Shortly thereafter broad screening of feed and food was initiated, the chemical structures of the major aflatoxins (B1, B2, G1, and G2) were elucidated, and research was conducted to prevent post-harvest contamination of grain crops through the modulation of storage conditions (Asao et al., 1965; Trenk and Hartman, 1970). However, it was found during a particularly severe outbreak of aflatoxin contamination in maize in the late 1970s in the U.S. that it was possible for *A. flavus* to both colonize and produce aflatoxin on developing maize kernels prior to harvest (Diener et al., 1983, 1987).

Since the extensive losses from the 1977 growing season due to aflatoxin contamination (Diener et al., 1983), research efforts have focused on determining the source of host plant resistance to prevent *A. flavus* colonization and subsequent aflatoxin production pre-harvest and before transportation to storage. These efforts have employed numerous techniques and approaches including modern plant breeding and genetics tools such as proteomic, transcriptomic, and biochemical analyses in an effort to discover the underlying mechanism of host plant resistance and the interaction between the two organisms. To date, these efforts have revealed that resistance is quantitatively inherited with a strong genotype by environment component. It is a complex interaction with a high degree of environmentally induced variability with abiotic and biotic stress strongly influencing resistance or susceptibility. Here we review the results of research into the mechanism of host resistance to both *A. flavus* colonization and abiotic stress, and propose future research directions for determining the relationship between oxidative stress and aflatoxin contamination.

## HOST RESISTANCE AGAINST *A. flavus*: GENE-FOR-GENE VS. GENOTYPE × ENVIRONMENT INTERACTION?

From a gene-for-gene perspective, host resistance against *A. flavus* colonization and subsequent aflatoxin contamination has been approached as a single virulence factor produced by the invading pathogen that would be countered by a single avirulence or resistance protein in the host. This results in compatible or incompatible reactions based on specific recognition (Keen, 1990). The particular virulence mechanisms of these plant-microbe interactions are utilized to classify plant pathogens into groups such as biotrophic, necrotrophic, and hemibiotrophic pathogens (Glazebrook, 2005).

The classification of *A. flavus* into a particular class of plant pathogens has yet to be determined. A microscopy study of the growth of *A. flavus *in maize kernel tissue by Smart et al. (1990) showed that cellular components such as cell walls were broken down in advance of mycelia. This has been interpreted as being indicative of necrotrophic pathogenicity in the literature (Mideros et al., 2009), however, not all research concurs with this conclusion. Magbanua et al. (2007) found that the colonization of kernel tissue from resistant maize lines exhibited increased levels of salicylic acid (SA) and unchanged levels of jasmonic acid (JA). Such patterns are commonly associated with resistance to biotrophic pathogens in various plant species (Glazebrook, 2005). As a facultative parasite, which naturally exists as a saprophyte, *A. flavus* may possess a unique pathogenicity mechanism that does not categorically fit into this classification scheme.

In keeping with the concept of gene-for-gene resistance, virulence factors produced by *A. flavus* during the colonization of maize tissues as well as maize kernel avirulence proteins have been the focus of multiple studies. These efforts have helped to better characterize the nature of this plant–pathogen interaction, and have sought to identify pathogen and host-derived proteins encoded by potential single-gene sources of host resistance or susceptibility. Proteomics-based techniques have identified several virulence proteins produced by *A. flavus*, most of which being hydrolytic enzymes. Examples of these enzymes are amylases, cellulases, chitinases, cutinases (e.g., phyto-cutinase), lipases, pectinases (P2c), proteases such as alkaline protease, and xylanases (Cleveland and Cotty, 1991; Guo et al., 1995; Chen et al., 1998, 1999; Fakhoury and Woloshuk, 1999; Mellon et al., 2000; Brown et al., 2001; Cleveland et al., 2004; Chen et al., 2009a; Pechanova et al., 2013). These enzymes are consistent with the biology and classification of *A. flavus* as a saprophyte since they typically catabolize decaying plant materials as a source of nutrition.

Several constitutively expressed and inducible proteins have been described in the literature, which have been shown to counter the function of other hydrolytic virulence proteins produced by *A. flavus*. For example, Chen et al. (1998) described a 14-kDa trypsin inhibitor (TI) which functions as an inhibitor of α-amylase, a protein utilized by *A. flavus* for the catabolism of complex carbohydrates. The same group also showed that silencing the expression of the TI gene in maize increases the susceptibility of maize kernel tissue to *A. flavus* infection and aflatoxin contamination (Chen et al., 2009b). In addition, β-1,3-glucanases, chitinases, pathogenesis-related proteins 10 and 10.1, ribosome inactivating proteins (RIPs), and zeamatin have also been shown to be involved in the resistance of maize against *A. flavus* (Mauch et al., 1988; Walsh et al., 1991; Huynh et al., 1992; Guo et al., 1997; Lozovaya et al., 1998; Chen et al., 2006; Chen et al., 2010; Xie et al., 2010). In peanut (*Arachis hypogaea*), Liang et al. (2005) reported that the increase of β-1,3-glucanases among resistant lines was higher than in the susceptible lines after infection with *A. flavus*.

Accumulation of such antifungal and avirulence proteins has been shown to contribute to the resistance observed in several maize lines along with morphological characteristics of resistant kernels such as thickened wax cuticles (Guo et al., 1995). Various breeding techniques have been employed to develop varieties with enhanced resistance to *A. flavus* and aflatoxin contamination on the basis of utilizing phenotypic screenings and molecular markers associated with known avirulence genes to identify quantitative trait loci (QTL) for selection (Brown et al., 2013). These efforts have met with some success such as in the case of Willcox et al. (2013) who identified 20 QTL explaining 22–43% of phenotypic variation within a F_2_ mapping population derived from Mp313E × Va35. This study, along with others (Paul et al., 2003; Brooks et al., 2005; Kelley et al., 2012), illustrate two important challenges in breeding resistant maize varieties, including: (1) that resistance to *A. flavus* is a quantitative trait involving multiple genes rather than single-gene forms of gene-for-gene resistance, and (2) a large genotype x environment (G × E) interaction conditions the expression of this trait. For example Willcox et al. (2013) determined that only 11 of the 20 identified QTL were consistently expressed across different environments with these accounting for 2.4–9.5% of phenotypic variance.

Environmental factors can have a significant effect on maize resistance to *A. flavus* and aflatoxin production, particularly abiotic stresses such as drought and heat stress. This was clearly demonstrated as early as 1977 when widespread and intense drought conditions in the Midwestern and Southeastern United States resulted in a high degree of aflatoxin contamination of maize kernels pre-harvest (Zuber and Lillehoj, 1979; Diener et al., 1987). However, maize genotypes possessing drought tolerance, such as Lo964 and Tex6, tend to be less susceptible to aflatoxin contamination (Guo et al., 2008; Luo et al., 2008; Jiang et al., 2012).

## RELATIONSHIP OF DROUGHT TOLERANCE, AFLATOXIN CONTAMINATION AND *A. flavus* RESISTANCE

It has been hypothesized that there is an underlying relationship among various molecular mechanisms of drought stress adaptation and resistance to *A. flavus* infection and subsequent aflatoxin contamination. This connection has been demonstrated experimentally in a number of studies. Chen et al. (2007) found a number of drought stress-related proteins that were induced in response to *A. flavus* colonization in maize endosperm tissue including late embryogenesis abundant proteins LEA 3, 14, peroxiredoxin (PER1), and a 17.9-kDa heat shock protein (HSP17.9). Pechanova et al. (2011) found that these proteins along with several antioxidant proteins, such as ascorbate peroxidase and superoxide dismutase (SOP), were up-regulated in resistant maize rachis tissue earlier in development and formed the basis, when combined with increased expression of anti-fungal and pathogenesis-related proteins, for resistance to *A. flavus* colonization.

It has also been shown that global defense regulators have the potential to modulate maize resistance to drought stress, oxidative stress, and possibly *A. flavus* resistance. The WRKY transcription factors have been shown to regulate the responses of multiple plant species to both biotic and abiotic stresses (Rushton et al., 2010). The transcription factor *Zm*WRKY33 was recently shown to enhance abscisic acid (ABA) signaling and enhance osmotic stress tolerance in maize seedlings (Li et al., 2013). *Zm*WRKY33 along with *Zm*WRKY19, whose homolog in *Arabidopsis thaliana *(*At*WRKY53) is known to induce a response to oxidative stress and regulate the expression of antioxidant enzymes like catalase (Miao et al., 2004; Eulgem and Somssich, 2007; Miao et al., 2007; Rushton et al., 2010). These factors were found to be up-regulated earlier in resistant maize varieties in response to *A. flavus* inoculation in whole kernel tissues (Fountain et al., 2013).

## OXIDATIVE RESPONSES OF PLANTS TO HERBIVORY

Oxidative responses of plants to feeding damage by both chewing and piercing-sucking insects and nematodes have been described in recent years. Walling (2000) and Kaloshian and Walling (2005) described the piercing-sucking hemipteran insect feeding on crop plants as resembling plant pathogen infections that they are often associated with chitosan (oligogalacturonides produced by pectinases) and reactive oxygen species (ROS)-activated wound or defense signaling pathways in host plants. Kessler and Baldwin (2002) reviewed the molecular mechanisms underlying plant responses to insect herbivory and how they differ from pathogen infections. They concluded that insect herbivores are physiologically independent from their host plants, whereas pathogens are physiologically dependent on their host plants for their growth and development. Oxidative enzyme-mediated wounding responses also play a critical role in understanding plant responses to insect herbivory, although the insect-specific elicitors frequently modify the responses of their host plants, and allow the host plants to optimize their defenses against a specific insect pest (Kessler and Baldwin, 2002). Das et al. (2008) studied the accumulation of ROS in cowpea–root-knot nematode interaction and confirmed that the induction of resistance is relatively late in this system. Typically, hypersensitive response is closely associated with an oxidative burst in infected tissue.

Bi and Felton (1995) reported that corn earworm (*Helicoverpa zea *Boddie), herbivory caused significant increases in lipid peroxidation and hydroxyl radical formation in the soybean leaves. The activities of several oxidative enzymes (i.e., lipoxygenases, peroxidase, diamine oxidase, ascorbate oxidase, and NADH oxidase I) were also increased following *H. zea* herbivory on soybean (*Glycine max*) leaves. They concluded that oxidative responses in the soybean plants may have led to a decrease in herbivory and an increase in oxidative damage to the plant. Ni et al. (2000) described the salivary enzyme profiles of the leaf-chlorosis-eliciting Russian wheat aphid (*Diuraphis noxia *Mordvilko), and the non-leaf-chlorosis-eliciting bird cherry-oat aphid (*Rhopalosiphum padi *L.), which differ in oxidative enzyme activities. While only peroxidase activity was detected in *R. padi*, catalase activity was only detected in *D. noxia.* The oxidative responses of four cereal plants (i.e., susceptible “Arapahoe” and resistant “Halt” wheat, susceptible “Morex” barley, and resistant “Border” oat) to the feeding of the two species of aphid differed (Ni et al., 2001a). The chlorosis-eliciting *D. noxia* feeding caused a three-fold increase in peroxidase activity in the resistant Halt wheat, and nine-fold increase in the susceptible Morex barley 9 days after infestation when compared to the control leaves. In contrast, *R. padi* did not cause any changes in peroxidase activity in any of the cereal leaves. At the same time, *D. noxia* feeding did not elicit any change in either catalase or polyphenol oxidase activity in comparison with either the *R. padi*-infested or the control cereal leaves (Ni et al., 2001a). Furthermore, oxidative bleaching in leaf chlorosis elicited by *D. noxia* was not detected, but Mg-dechelatase activity was increased in the *D. noxia*-elicited chlorosis in wheat leaves (Ni et al., 2001b).

Zavala et al. (2013) also proposed a cellular mechanism to decipher the influence of elevated CO_2_ on insect herbivory. They highlighted that oxidative enzyme activities in the sub-cellular organelles, such as peroxisomes and chloroplasts, via the jasmonic signaling pathway are likely to be critical factors in dissecting the molecular mechanisms of plant defenses against both biotic and abiotic stresses. In general, the oxidative responses of plants to pathogen infection and insect herbivory under varying environmental conditions (e.g., drought and the elevated CO_2_) are critical for the management of pest outbreaks and the reduction of mycotoxin contamination in agricultural crops.

## THE ROLE OF OXIDATIVE STRESS IN AFLATOXIN BIOSYNTHESIS

The presence of increased expression of antioxidant mechanisms in resistant maize tissues leads to the hypothesis that increased resistance to ROS-induced oxidative stress may correlate to resistance to *A. flavus* and aflatoxin contamination. Although this conclusion seems credible due to the high degree of correlative evidence present in the literature, the exact mechanism of how this phenomenon functions has yet to be elucidated completely. More recent studies into the biology of *A. flavus* and the mechanisms regulating the production of aflatoxin may illuminate this issue (Magbanua et al., 2007; Roze et al., 2013).

Aflatoxin biosynthesis is a complex process involving multiple gene products and regulatory mechanisms coded for by an approximately 70-kb cluster of 25 genes (Yu et al., 2004). This pathway is responsible for the biosynthesis of five major mycotoxins: sterigmatocystin, and aflatoxins B_1_, B_2_, G_1_, and G_2_ (Yu et al., 2004), and is the focus of intensive research into methods of negatively regulating its function. Although the structure and biochemical characteristics of aflatoxins have been known since the 1960s (Asao et al., 1965; Wogan, 1966), the specific purpose of their production by *A. flavus* or other aflatoxigenic fungi has remained a mystery. Prior research exploring the roles of oxidative stress in regulating aflatoxin biosynthesis as well as recent discoveries into the upstream regulation of major pathway regulatory factors have begun to elucidate the biological function of aflatoxins (Roze et al., 2013). It has been shown that aflatoxin production by *A. flavus* is higher in maize kernel tissues containing higher levels of lipids, such as embryo tissues (Earle et al., 1946; Fabbri et al., 1980; Brodhagen and Keller, 2006). The roles of lipids in regulating aflatoxin biosynthesis in *Aspergillus spp*. have been investigated, particularly oxylipins (Reviewed in Gao and Kolomiets, 2009). Earlier research by Fabbri et al. (1983) found that seeds of high-oil crops such as peanut support higher levels of aflatoxin production by *A. parasiticus* than seeds of graminaceous plants such as maize or wheat, which contain higher levels of starch. In addition, they found that culturing *A. parasiticus* amended with peroxidized lipids resulted in significantly elevated aflatoxin production with no significant effect on fungal biomass (Fabbri et al., 1983).

Lipid peroxidation is a byproduct of lipid metabolism in peroxisomes as well as the reaction of naturally produced free fatty acids with ROS (Reverberi et al., 2012). Therefore, it is possible that excessive peroxisome function in the fungal mycelia or oxidative stress may be a causative factor in the production of aflatoxin. *In vitro*, Jayashree and Subramanyam (2000) showed that toxigenic strains of *A. parasiticus* have increased oxygen requirements, which they postulate to be a potential source of ROS accumulation, in comparison to non-toxigenic strains. In addition, they showed that higher levels of glutathione and thiobarbituric acid-reactive substances (TBARS), as well as antioxidant enzyme activities, were present in toxigenic strains in comparison to non-toxigenic strains. This indicated that oxidative stress may be a pre-requisite for aflatoxin production (Jayashree and Subramanyam, 2000). Also, Reverberi et al. (2012) found that bezafibrate and transformation of the *Cymbidium ringspot virus* P33 gene into *A. flavus* induced peroxisome proliferation resulting in an increase in aflatoxin production both *in vitro* and when cultured on maize kernel tissues in addition to increased levels of antioxidant enzyme gene expression, lipid metabolism, oxylipin biosynthesis, and ROS accumulation in the *A. flavus* mycelia. Recent studies have also found that cAMP and G-protein-mediated quorum sensing signaling pathways based on oxylipin perception can play a vital role in growth regulation and aflatoxin production in *A. nidulans* (Affeldt et al., 2012). Such G-protein mediated signaling has also been reported in other *Aspergillus spp*. including *A. fumigatus* (Grice et al., 2013). Therefore, it may be concluded that oxidative stress in *A. flavus* induced by ROS and/or oxylipins in the growth environment/medium will result in increased aflatoxin production from a biochemical perspective.

Recent studies have also shown that ROS can play a role in the transcriptional regulation of aflatoxin and sterigmatosystin biosynthesis pathway genes. Reverberi et al. (2008) found that a putative binding site for the Ap*yapA* gene, which regulates oxidative stress tolerance and conidiogenesis, was present in the promoter of the regulatory gene *aflR* in *A. parasiticus*, and that silencing Ap*yapA* results in an increase in aflatoxin biosynthesis. It has also been shown that the basic leucine zipper (bZIP) transcription factor AtfB regulates the aflatoxin biosynthesis genes *fas-1*, *ver-1*, and *omtA* as well as antioxidant genes encoding for catalase and SOP (Hong et al., 2013). They also found that the promoter regions associated with AtfB also contained cAMP-responsive elements implicating cAMP in the regulation of aflatoxin biosynthesis (Hong et al., 2013).

## POTENTIAL REACTIVE OXYGEN SPECIES-MEDIATED CROSSTALK BETWEEN MAIZE AND *A. flavus*

Given the apparent role of oxidative stress in the promotion of aflatoxin biosynthesis, the hypothesis has been proposed that aflatoxin may function as a form of antioxidant protection to Aspergilli (Reverberi et al., 2010). This would provide an explanation to the long standing question, rather the mystery, as to the biological significance of aflatoxin, although the potential antioxidant mechanism of action of aflatoxin has yet to be fully elucidated. In addition, this explanation also provides for the potential role of ROS and oxylipins cross-kingdom communication between maize and *A. flavus*.

Cross-kingdom communication between plants and various fungi through the use of oxylipins has been previously documented (Eckardt, 2008; Christensen and Kolomiets, 2011). Specifically, the role of oxylipins has been clearly illustrated in the specific interaction between maize and *A. flavus* (Gao and Kolomiets, 2009). In a recent study, it was found that maize lipoxygenase-3 (LOX3) is required for resistance to *A. flavus* indicating that certain 9-oxylipins can play important roles in suppressing aflatoxin biosynthesis while other oxylipins may promote aflatoxin biosynthesis (Gao et al., 2009). The role of LOX-1 in resistance mechanisms against *A. flavus*, *A. nidulans, *and *A. parasiticus* has also been implied in soybean (Doehlert et al., 1993; Burow et al., 1997), although there are contradicting reports on the subject in the literature. Mellon and Cotty (2002) found that soybeans lacking LOX activity were just as resistant to *A. flavus* and aflatoxin contamination as those possessing LOX activity. This seems to imply some degree of specificity in the role of LOX enzymes or their products in resistance to certain *Aspergillus* species.

In addition to oxylipins, other host-derived compounds may influence oxidative stress including ROS and phytohormones. It was found that 2-chloroethyl phosphoric acid (CEPA), the metabolic precursor to ethylene, was capable of reducing the expression of *aflR* and *aflD* (two key genes in the aflatoxin biosynthetic pathway), reducing the accumulation of oxidative compounds, and regulating glutathione redox in *A. flavus* mycelia (Huang et al., 2009). Therefore, host-derived ethylene may result in the reduction of ROS accumulation in *A. flavus* mycelia and reduce aflatoxin biosynthesis. This hypothesis seems plausible since the expression of the maize Ethylene Responsive Factor 1 (*Zm*ERF1), a key transcription factor involved in ethylene and JA signaling, was found to be higher in the immature kernel tissues of the resistant maize inbred TZAR101 (Menkir et al., 2008) in comparison to the susceptible maize inbred B73 following *A. flavus* inoculation (Fountain et al., 2013).

Previous research has shown that maize varieties resistant to *A. flavus* tend to accumulate antioxidant enzymes, such as peroxidase and SOP, and tend to be more resistant to drought and heat stress than varieties susceptible to *A. flavus* (Guo et al., 2008; Pechanova et al., 2011). Given the reported role of oxidative stress in *Aspergillus spp*. biology, it may be possible for host-derived antioxidant proteins, phytohormones, and oxylipins to negatively regulate the production of aflatoxin in infecting *A. flavus* by reducing the level of oxidative stress endured by both the host and the fungus, particularly during drought or heat stress. Such an interaction may explain several observed phenomena in the literature. For example, Guo et al. (1996, 1997) observed that the pre-incubation of maize kernels in high-humidity conditions for 3 days prior to inoculation with *A. flavus *results in a significant reduction in aflatoxin contamination in comparison with kernels inoculated without pre-incubation. Given the fact that ROS such as hydrogen peroxide accumulates to maximum quantities two days post-imbibition (DPI) followed by increased catalase activity beginning at three DPI in maize kernels (Hite et al., 1999), a combination of host-derived resistance and antioxidant proteins and reduced ROS production at the time of inoculation may have contributed to the reduction in aflatoxin production (Guo et al., 1996).

## CONCLUSION

Determining the role of oxidative stress in the regulation of aflatoxin biosynthesis as well as the role of host defenses against both *A. flavus* infection and mycotoxin biosynthesis are critical areas of research for the mitigation of aflatoxin contamination in maize. Maize resistance to *A. flavus* is a complex, quantitative trait which is the culmination of the interaction of numerous resistance-associated proteins and antioxidant enzymes which have been the subject of more than 50 years of rigorous research. Solutions to the problem of aflatoxin contamination of crops, particularly maize, have been elusive given the high level of environmental influence on the interaction and the lack of stable resistance in maize germplasm across multiple environments. By better understanding the role of oxidative stress and its remediation by the host and the pathogen, additional tools will be made available to counter the threat aflatoxin poses to food safety and security and further enhance the knowledge of cross-kingdom interactions which may be applied to other mycotoxin producing pathogens in various agricultural commodities.

## Conflict of Interest Statement

The authors declare that the research was conducted in the absence of any commercial or financial relationships that could be construed as a potential conflict of interest.
